# Non-sentinel node metastasis prediction during surgery in breast cancer patients with one to three positive sentinel node(s) following neoadjuvant chemotherapy

**DOI:** 10.1038/s41598-023-31628-2

**Published:** 2023-03-18

**Authors:** Jung Whan Chun, Jisun Kim, Il Yong Chung, Beom Seok Ko, Hee Jeong Kim, Jong Won Lee, Byung Ho Son, Sei-Hyun Ahn, Sae Byul Lee

**Affiliations:** Division of Breast Surgery, Department of Surgery, Asan Medical Center, University of Ulsan College of Medicine, 88, Olympic-ro 43-gil, Songpa-Gu, Seoul, 05505 Republic of Korea

**Keywords:** Cancer, Oncology

## Abstract

Our aim was to develop a tool to accurately predict the possibility of non-sentinel lymph node metastasis (NSLNM) during surgery so that a surgeon might decide the extent of further axillary lymph node dissection intraoperatively for patients with 1–3 positive sentinel lymph node(s) (SLN) after neoadjuvant chemotherapy. After retrospective analysis of Asan Medical Center (AMC) database, we included 558 patients’ records who were treated between 2005 and 2019. 13 factors were assessed for their utility to predict NSLNM with chi-square and logistic regression with a bootstrapped, backward elimination method. Based on the result of the univariate analysis for statistical significance, number of positive SLN(s), number of frozen nodes, Progesterone Receptor (PR) positivity, clinical N stage were selected for the multivariate analysis and were utilized to generate a nomogram for prediction of residual nodal disease. The resulting nomogram was tested for validation by using a patient group of more recent, different time window at AMC. We designed a nomogram to be predictive of the NSLNM which consisted of 4 components: number of SLN(s), number of frozen nodes, PR positivity, and clinical N stage before neoadjuvant chemotherapy. The Area under the receiver operating characteristics curve (AUC) value of this formula was 0.709 (95% CI, 0.658–0.761) for development set and 0.715 (95% CI, 0.634–0.796) for validation set, respectively. This newly created AMC nomogram may provide a useful information to a surgeon for intraoperative guidance to decide the extent of further axillary surgery.

## Introduction

Although previous trials of the survival benefit for patients who underwent neoadjuvant chemotherapy (NAC) failed to prove relative superiority over patients who treated with adjuvant chemotherapy, the addition of NAC for treatment of eligible breast cancer patients have been widely accepted. NAC is helpful in reducing the need for total mastectomy, full axillary lymph nodal dissection (ALND) with its associated morbidity without increasing loco-regional recurrence^1^. Although we admit that more clinical data will be needed to definitely confirm the feasibility of the procedure in post-NAC setting, sentinel lymph node biopsy (SLNB) after NAC for patients with clinically positive axilla resulted in acceptable accuracy, making it one of the effective axillary management strategy^1–3^. Despite of the fact that the feasibility of SLNB in the post-NAC setting in either clinically node-negative or node-positive patients has been an evolving area of active debate, our institutional practice pattern has also followed to incorporate SLNB as the initial approach for the axilla after NAC unless the patient had significant disease burden remained or progressive disease.

According to the recent National Comprehensive Cancer Network (NCCN) guidelines, for patients who presented with node-positive breast cancer after NAC, completion ALND has been the standard surgical management^4^.

Among breast cancer patients with positive sentinel lymph nodes (SLNs) who have received axillary lymph node dissection (ALND), the non-sentinel lymph nodes (NSLNs) have shown to be tumor free in substantial portion of patients. Dingemans et al. reported that 59% of primary breast cancer patients were without NSLNM among 160 patients with macrometastasis to SLNs. These patients have been treated with unnecessary ALND with significant possible complications and without therapeutic benefits^5^. In an analysis by Jeruss et al., their study population included 104 patients who received NAC, had a positive SLN, and underwent ALND between 1997 and 2005^6^. 44% of their research cohort did not have positive non-SLNs. They analyzed factors predicting additional NSLNM in which included lympho-vascular invasion (LVI), method of SLN metastasis detection, multicentricity, ALN status at presentation and pathological tumor size. Based on this result, they derived the MD Anderson nomogram with a significant AUC. Also, a study by Gimbergues et al. 132 patients were followed prospectively between 2001 and 2007. All patients received NAC and underwent SLN biopsy with ALND level I and II^7^. They reported that 47.1% of their patient population did not have NSLNM and they tested the accuracy of previous nomograms from the Memorial Sloan-Kettering Cancer Center, the MD Anderson Cancer Center, and the Tenon Hospital in Paris with their AUC result between 0.7 and 0.8.

However, these nomograms are mostly based on factors from a final pathology report after surgery which included lympho-vascular invasion (LVI), pathologic tumor size, and size of SLN metastasis, etc. Thus, when indicated, patients are supposed to undergo further axillary dissection on a separate schedule. In this study, we retrospectively analyzed patients’ data from Asan Medical Center (AMC) to develop a nomogram that might help predict the possible NSLNM based on the clinical informations available before a planned surgery.

## Material and methods

### Patients

We reviewed the data of patients who underwent breast surgery with ALND after neoadjuvant chemotherapy between 2005 and 2019. We included patients with 1–3 metastasis-positive sentinel node(s) who were treated with standard axillary procedure. All included patients were treated with a full course of standard neoadjuvant therapy according to the direction of the oncologists in AMC. As a result of ALND, we could identify the patients with or without residual nodal disease based on the final pathology reports. We excluded the patients with bilateral breast cancer, inflammatory breast cancer or presence of distant metastasis at presentation, those with more than 4 sentinel nodes positive for metastasis, who proceeded directly to ALND without SNB or SNB only. We also excluded the patients whose neoadjuvant chemotherapy were incomplete due to patients’ intolerance or refusal. Finally, a total of 558 patients were included for further analysis. 384 patients’ records who were treated between 2005 and 2016 were utilized for the development of prediction model and the data of 174 patients who were treated from 2017 to 2019 were used for validation of the generated prediction model.

The patients’ data were reviewed for the total number of metastatic nodes on the final pathology report, intraoperative frozen section biopsy result of sentinel node(s), presence or absence of additional metastatic non-sentinel nodes and number, tumor invasion depth, tumor biology, initial clinical stage before chemotherapy, radiology report of ultrasonogram or MRI of breast before and after chemotherapy. Because the study was based on retrospective clinical data, the need for informed consent was waived. And this study and the waiver were approved by the Asan Medical Center Institutional Review Board, Seoul, South Korea. (20,171,341).

### Neoadjuvant chemotherapy and SNLB mapping method

Neoadjuvant chemotherapy was administered to a patient every 3 weeks, and a regimen was selected among standard proposed regimens based on the clinical stage or tumor biology of a patient. Although the standard regimens evolve continuously, the oncologists of our institution generally followed the most updated NCCN guideline of the time. Surgery was performed at 3 to 4 weeks after the completion of NAC. We assessed a patient’s response to neoadjuvant chemotherapy by using either ultrasonogram or MRI of breast before and after treatment. According to the Revised Response Evaluation Criteria in Solid Tumors (RECIST guideline, version 1.1), we defined partial remission when more than 30 percent decrease in the sum of the longest diameter of the target lesions compared with baseline. Also, we used the term complete remission when we found disappearance of all target lesions. All tumors that did not meet the above criteria were classified as stable disease.

We used ^99m^Tc-sulfur colloid diluted in normal saline for radiopharmaceutical agent with gamma probe detection (NeoProbe2000, US surgical, Norwalk, CT) for SLN identification. We injected the mapping agent periareolarly and the breast was massaged for 5 min. Along with the most radioactive nodes, clinically enlarged, firm or palpable axillary lymph nodes without active gamma signal were also excised and were included with total number of SLNs.

### Statistical analysis

The clinicopathologic factors for baseline patient characteristics and for comparison of the training and validation group were divided into categorical and continuous variables. The independent t-test was used to compare continuous variables, and the chi square test and Fisher’s exact test were utilized to compare categorical variables in order to generate *p*-values. In order to identify significant factors to predict a possibility of residual disease of non-sentinel nodes, we included initially the following parameters for univariate analysis; age at diagnosis, tumor grade, hormone receptor score, HER2 status, classification into 4 subtypes (HR+/HER2−, HR+/HER2+, HR−HER2+, HR−/HER2−), Ki-67, clinical T stage and N stage before neoadjuvant chemotherapy and its degree of response to a therapy, number of metastatic sentinel nodes, total number of submitted sentinel nodes for frozen section biopsy, the greatest tumor invasion depth of in the sentinel nodes.

We used 384 patients’ data who were treated between 2005 and 2016 for the development of prediction model and the data of 174 patients who were treated from 2017 to 2019 for validation. In the development set, univariate assessment of these factors was performed using a logistic regression model. A multivariable logistic regression model was used to further analysis and to generate a prediction model for the possibility of residual nodal disease after neoadjuvant chemotherapy when 1–3 sentinel nodes were positive intraoperatively. The multivariable model was built with the predictors selected in more than 50% of 1000 bootstrap resamples using backward elimination. The final model was estimated with penalized maximum likelihood and presented as a nomogram. The discrimination ability of the nomogram was assessed by using area under the receiver operating characteristic curve (AUC). The calibration ability was assessed by using calibration plot and Hosmer–Lemeshow test. We ran an internal validation using bootstrapping with 1000 iterations and calculated optimism-corrected AUC (C statistics). In validation set, the discrimination and calibration ability were also evaluated. All tests were 2-sided, and *p*-value of less than 0.05 was considered statistically significant. Statistical analysis was conducted with SPSS statistics version 23.0 (IBM Corp., Armonk, USA) and R (version 3.6.1; R Foundation for Statistical Computing, https://www.R-project.org).

### Ethical approval

All procedures involving human participants were performed in accordance with the ethical standards of the institutional and/or national research committee and with the 1964 Helsinki Declaration and its later amendments or comparable ethical standards.

## Results

### Baseline characteristics

Demographics for the 558 patients whose clinical data was used for developing the nomogram are presented in Table 1. In total, the majority of patients were less than 50 years (63.6%), had a single SLN metastasis at the time of surgery (50.9%), had three to five frozen biopsy were sent for pathologic confirmation of SLN status (66.1%), had clinical T stage 2 (63.4%). Also, a substantial portion of our patients had N1 stage disease both before (74.4%) and after (75.1%) NAC. The majority of tumors were low grade (81%), estrogen receptor positive (80.6%), progesterone receptor positive (66.5%), HER2 negative (78.3%), biological subtype of hormone receptor positive and HER2 negative (78.3%). As shown in the Table 1, there were significant differences in the baseline characteristics of patients who had or did not have residual nodal disease. The residual nodal disease group had a higher rate of estrogen receptor-positive patients (85.7%, *p* = 0.007) and a higher rate of progesterone receptor-positive patients (72.2%, *p* = 0.011) than the no nodal residual disease group. Also, patients with nodal residual disease had a higher initial N stage (*p* < 0.001), large number of positive SLN (*p* < 0.001), a higher pathologic T stage (*p* = 0.001), and a higher N stage (*p* < 0.001) compared with those who having no nodal residual disease. For the type of surgery, 53.8% of patients underwent total mastectomy and 46.2% underwent breast conserving surgery that was not significantly different between subgroups with or without residual nodal disease. (*p* = 0.079).

**Table 1:** Baseline patient characteristics and association between residual disease and clinicopathologic variables.

	Total	Residual disease		*p*-value
		no	yes	
Number of patients	558	313	245	
Age				0.035
< 50 years	355 (63.6)	211 (67.4)	144 (58.8)	
≥ 50 years	203 (36.4)	102 (32.6)	101 (41.2)	
Tumor grade				0.002
G1/2	452 (81.0)	242 (77.3)	210 (85.7)	
G3	102 (18.3)	71 (22.7)	31 (12.7)	
Unknown	4 (0.7)	0 (0.0)	4 (1.6)	
Estrogen Receptor				0.007
Negative	108 (19.4)	73 (23.3)	35 (14.3)	
Positive	450 (80.6)	240 (76.7)	210 (85.7)	
Progesterone Receptor				0.011
Negative	187 (33.5)	119 (38.0)	68 (27.8)	
Positive	371 (66.5)	194 (62.0)	177 (72.2)	
HER2 status				0.393
Negative	437 (78.3)	241 (77.0)	196 (80.0)	
Positive	121 (21.7)	72 (23.0)	49 (20.0)	
Biological Subtype				0.039
HR+/HER2−	379 (67.9)	199 (63.6)	180 (73.5)	
HR+/HER2+	72 (12.9)	41 (13.1)	31 (12.7)	
HR−/HER2+	44 (7.9)	30 (9.6)	14 (5.7)	
HR−/HER2−	63 (11.3)	43 (13.7)	20 (8.2)	
Initial T stage				0.165
1	54 (9.7)	32 (10.2)	22 (9.0)	
2	354 (63.4)	208 (66.5)	146 (59.6)	
3	138 (24.7)	66 (21.1)	72 (29.4)	
4	12 (2.2)	7 (2.2)	5 (2.0)	
Initial N stage				< 0.001
0	92 (16.5)	65 (20.7)	27 (11.0)	
1	415 (74.4)	229 (73.2)	186 (75.9)	
2	51 (9.1)	19 (6.1)	32 (13.1)	
Response to NAC				0.891
Complete remission	22 (3.9)	14 (4.5)	8 (3.3)	
Partial remission	406 (72.8)	228 (72.8)	178 (72.7)	
Stable disease	119 (21.3)	65 (20.8)	54 (22.0)	
Progressive disease	11 (2.0)	6 (1.9)	5 (2.0)	
Surgery type				0.079
Breast conserving surgery	258 (46.2)	155 (49.5)	103 (42.0)	
Mastectomy	300 (53.8)	158 (50.5)	142 (58.0)	
Number of positive SLNs				< 0.001
1	284 (50.9)	185 (59.1)	99 (40.4))	
2	203 (36.4)	106 (33.9)	97 (39.6)	
3	71 (12.7)	22 (7.0)	49 (20.0)	
Pathologic T stage				0.001
0	29 (5.2)	18 (5.8)	11 (4.5)	
1	235 (42.1)	150 (47.9)	85 (34.7)	
2	238 (42.7)	124 (39.6)	114 (46.5)	
3	56 (10.0)	21 (6.7)	35 (14.3)	
Pathologic N stage				< 0.001
1	419 (75.1)	309 (98.7)	110 (44.9)	
2	139 (24.9)	4 (1.3)	135 (55.1)	

SLN (Sentinel Lymph Node).

NAC (Neoadjuvant chemotherapy).

Response to NAC: followed the Revised Response Evaluation in Solid Tumors (RECIST) guideline, version 1.1.

Clinicopathologic information of patients according to the training and validation groups were presented in the Table 2. The median age of the test population was 46.4 years (range: 24–74), and 48.3 years (range: 26–78) for the validation group. The hormone receptor status and HER2 positivity did not significantly differ between the two groups. The mean clinical tumor size before NAC was 44.2 mm (Std. Deviation 20.3 mm) for the training population and it was 38.2 mm (Std. Deviation 18.7 mm) for the validation group. The mean pathologic tumor size after NAC was 21.4 mm (Std. Deviation 15.9 mm) for the training population (50.1% mean reduction) and it was 18.7 mm (Std. Deviation 14.2 mm) after NAC (50.2% mean reduction) for validation group, respectively. Clinical parameters of both groups of patients exhibited no significant difference except clinical N stage in which the proportion of N2 patients were 7.3% in the test group and 16.2% in the validation group. The rates of patients underwent total mastectomy or breast conserving surgery were not statistically different between the training group and the validation group. (*p* = 0.309) The number of positive SLN(s) at the time of surgery and the proportion of patients with residual nodal disease were critical parameters for developing the nomogram and its p values failed to show statistical difference. Comparison of detailed clinicopathologic factors between the training and validation group according to residual nodal disease was presented in the Supplementary Table 1.

**Table 2:** Comparison of the training group and validation group.

	Training group	Validation group	
Number of patients	384	174	*p*-value
Age at diagnosis			0.203
< 50 years	251 (65.4)	104 (59.8)	
≥ 50 years	133 (34.6)	70 (40.2)	
Tumor grade			0.849
G1/2	313 (81.5)	143 (82.2)	
G3	71 (18.5)	31 (17.8)	
Estrogen receptor			0.698
Negative	76 (19.7)	32 (18.4)	
Positive	308 (80.3)	142 (81.6)	
Progesterone receptor			0.475
Negative	125 (32.6)	62 (35.6)	
Positive	259 (67.4)	112 (64.4)	
HER2 status			0.545
Negative	298 (77.6)	139 (79.9)	
Positive	86 (22.4)	35 (20.1)	
Biological subtype			0.359
HR+/HER2−	260 (67.7)	119 (68.4)	
HR+/HER2+	49 (12.8)	23 (13.2)	
HR−/HER2+	35 (9.1)	9 (5.2)	
HR−/HER2−	40 (10.4)	23 (13.2)	
Clinical T stage			0.051
T1	32 (8.4)	22 (12.6)	
T2	245 (63.8)	109 (62.6)	
T3	95 (24.7)	43 (24.8)	
T4	12 (3.1)	0	
Clinical N stage			0.032
N0	70 (18.2)	22 (12.6)	
N1	286 (74.5)	129 (74.2)	
N2	28 (7.3)	23 (16.2)	
Surgery type			0.309
Breast conserving surgery	172 (44.8)	86 (49.4)	
Total mastectomy	212 (55.2)	88 (50.6)	
Number of positive SLN(s)			0.608
1	190 (49.5)	94 (54.0)	
2	144 (37.5)	59 (33.9)	
3	50 (13.0)	21 (12.1)	
Residual nodal disease			0.942
Absent	215 (56.0)	98 (56.3)	
Present	169 (44.0)	76 (43.7)	
Mean Invasion Depth in SLN (mm)	5.3 (0.3–30)	5.7 (0.3–23)	0.294
Response to NAC			0.128
Complete remission	18 (4.7)	4 (2.3)	
Partial remission (≥ 30% reduction)	269 (70.1)	137 (78.7)	
Stable disease	90 (23.4)	29 (16.7)	
Progressive disease	7 (1.8)	4 (2.3)	

SLN (Sentinel Lymph Node).

NAC (Neoadjuvant chemotherapy).

Response to NAC: followed the Revised Response Evaluation in Solid Tumors (RECIST) guideline, version 1.1.

### Factors for predicting NSLNM and development of a nomogram

**Figure 1:**
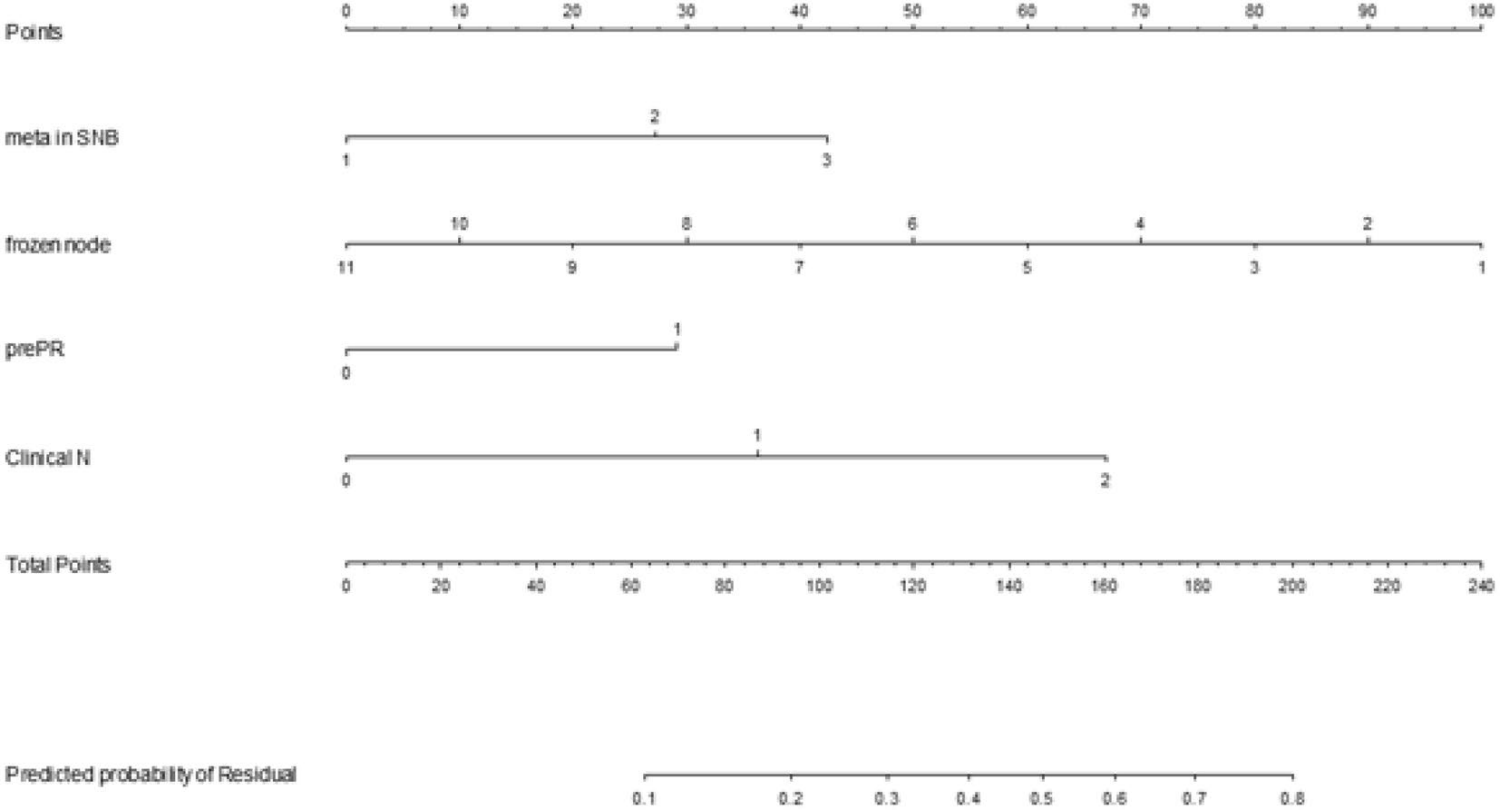
The nomogram to predict non-sentinel lymph node metastasis in breast cancer patients with 1–3 positive sentinel lymph node(s) on a frozen biopsy result.

**Figure 2:**
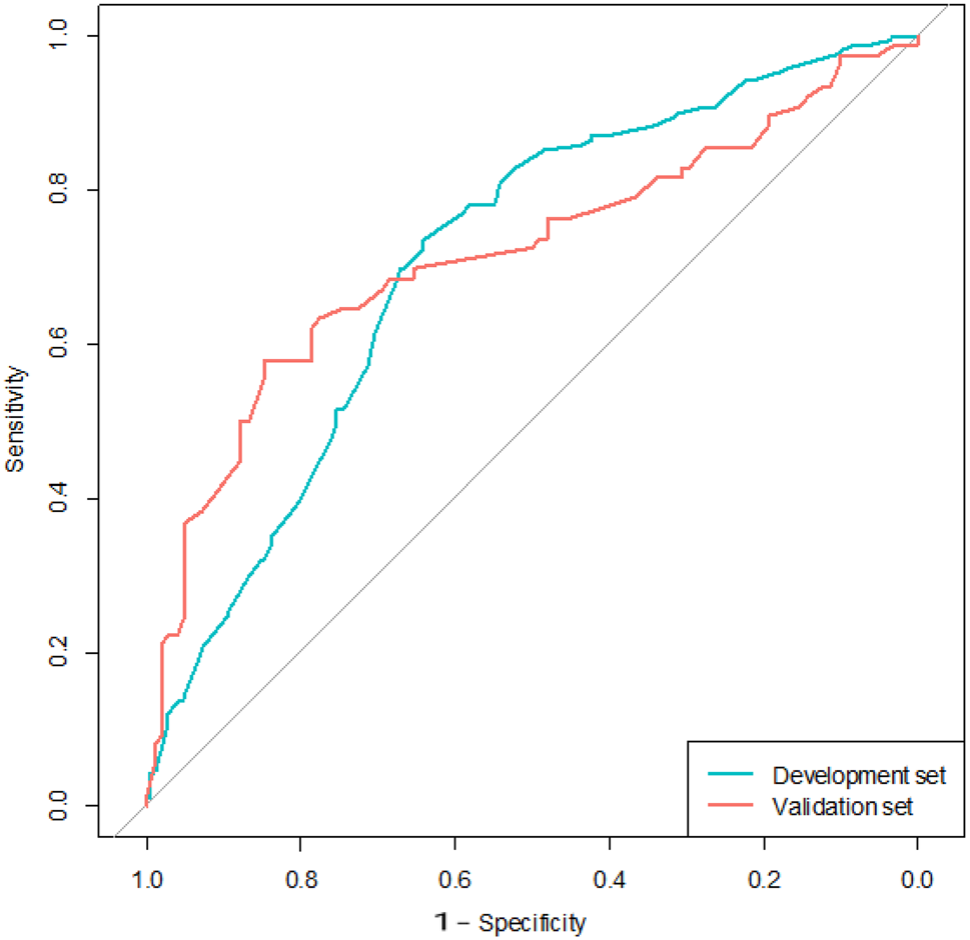
Receiver operating characteristic curve (ROC) of the nomogram. Area under the ROC curve was 0.709 on the development set and 0.715 on the validation set, respectively.

Our purpose was to develop a tool that can associate routinely measured clinical factors to the actual probability of NSLNM during surgery. Detailed analysis result was shown in the Table 3. On the univariate analysis, number of positive SLN(s), number of frozen nodes, tumor grade, ER and PR positivity, clinical N stage and biological subtype were found to be significantly associated with the possibility of residual nodal disease. The odds ratio showed notable increase in parallel to the number of positive SLN(s) and initial N stage. On multivariate stepwise logistic regression analysis, number of metastatic SLN(s), number of frozen SLN(s), PR positivity and preoperative clinical N stage were found to be the independent predictors of NSLNM. These four variables were included to develop the nomogram (Fig. 1). The Area under the receiver operating characteristics curve (AUC) values of this formula were 0.709 (95% CI, 0.658–0.761, Hosmer–Lemeshow test *p*-value 0.176) for the development set (Fig. 2 and Table 4). In order to test the discrimination ability of the nomogram, we performed an independent validation study with the data from a different cohort of 174 patients who underwent surgery at AMC from 2017 to 2019. The Area under the receiver operating characteristics curve (AUC) values of this formula were 0.715 (95% CI, 0.634–0.796, Hosmer–Lemeshow test *p*-value 0.104) for the validation set. Since the null hypothesis (H_0_) for this Hosmer–Lemeshow’s test was that this model was a good fit for the data, *p*-value exceeding 0.05 was interpreted that this prediction model was appropriate for explaining the data.

**Table 3:** Result of logistic regression for residual nodal disease.

Parameter		Univariable				Multivariable			
		Odds ratio	95% CI		*P* value	Odds ratio	95% CI		*P* value
Number of positive SLN(s)	1	1			< 0.001*	1			0.001
	2	1.836	1.180	2.856	0.007	2.040	1.279	3.256	0.003
	3	2.754	1.453	5.219	0.002	3.027	1.571	5.831	0.001
Number of frozen SLN(s)		0.848	0.747	0.963	0.011	0.770	0.670	0.885	0.000
Invasion depth of SLN(s)		1.047	0.995	1.103	0.079				
Age at diagnosis		1.005	0.983	1.028	0.645				
Tumor grade	1	1			0.040				
	2	1.491	0.350	6.348	0.589				
	3	0.748	0.164	3.412	0.708				
HER2 status	1	0.742	0.454	1.212	0.233				
Estrogen Receptor	0–2	1							
	> 2	2.241	1.301	3.860	0.004				
Progesterone Receptor	0–2	1				1			
	> 2	1.901	1.220	2.964	0.005	2.142	1.343	3.416	0.001
Biological subtype	1: LumA	1.892	0.935	3.831	0.076				
	2: LumB	1.997	0.845	4.718	0.115				
	3: HER2	0.615	0.220	1.723	0.355				
	4: Triple Negative	1			0.012				
Ki-67 status		0.995	0.987	1.003	0.238				
	> 20	1.136	0.712	1.813	0.593				
Initial T stage	1	1			0.112				
	2	1.149	0.538	2.457	0.719				
	3	2.016	0.886	4.585	0.095				
	4	1.190	0.308	4.604	0.801				
Initial N stage	0	1			< 0.001	1			< 0.001
	1	2.269	1.275	4.035	0.005	2.584	1.438	4.642	0.002
	2	5.667	2.187	14.683	0.000	5.743	2.240	14.722	0.000
Response to NAC	1 = 100%	1			0.958				
	2 = 30% ~ 99%	0.836	0.444	1.573	0.579				
	3 = 0% ~ 29%	0.851	0.417	1.738	0.658				
	4 = < 0%	0.818	0.164	4.072	0.806				

*The *p*-value written in the reference line means the overall *p*-value of the variable.

**Table 4:** Discrimination and Calibration ability of the developed nomogram.

		N	Residual	AUC		95% CI	Hosmer–Lemeshow test		
							X-squared	DF	*P* value
Training	2005–2016	384	169	0.709	0.658	0.761	8.957	6	0.176
Validation	2017–2019	174	76	0.715	0.634	0.796	10.535	6	0.104

AUC = C statistics.

Optimism corrected C statistics by 1000 bootstrap resamples.

## Discussion

Despite of traditional axillary surgery remains one of the standard management options, optimal treatment of the axilla has been an evolving area toward reducing its related morbidity. According to the recent NCCN guideline for invasive breast cancer, we are recommended to perform ALND level I, II or SLNB in selected cases when nodes clinically negative after NAC, if FNA or core biopsy positive before NAC was given^4^. ALND, as a means for achieving local disease control, carries an indisputable and often unacceptable risk of complications such as seroma, infection, and lymphedema^8^. However, 40% to 60% of patients who underwent ALND were actually without residual axillary disease according to the previous studies which analyzed patients with primary breast cancer or who underwent neoadjuvant chemotherapy^5–7^. In our study population, 56% of patients were revealed to have no residual nodal metastasis after completion of axillary dissection which was proceeded based on a positive sentinel nodal biopsy result at the time of surgery. As a result, a substantial portion of patients may have been exposed to the significant morbidity of the extensive axillary surgery without actual clinical benefit. Thus, our study purpose was to find a tool that allows a surgeon to be more selective in choosing a subgroup of patients who might be spared from the possible morbidity of ALND. Our nomogram is composed of four variables which included number of metastatic SLN(s), number of frozen nodes, PR positivity, and preoperative clinical N stage. These parameters were available before proceeding to a full ALND.

Several models have been proposed to predict the presence of NSLNM for breast cancer patients with or without NAC^6,9–13^. One of the most widely used nomogram was developed by Van Zee et al. which included 8 variables of pathological size, lymphovascular invasion, method of detection, number of positive SLNs, multifocality, and number of negative SLNs with statistical significance^13^. ER status and nuclear grade were included in the model but failed to show significant association with the likelihood of NSLNM. The overall discriminative ability of this nomogram, as measured by the ROC curve, was 0.76 for the retrospective population. The AUC value for the corresponding prospective population was 0.77. However, this model is only applicable to patients without NAC. Patients who were treated with NAC may need another version of nomogram for accurate prediction of NSLNM. Also, the variables such as pathological tumor size and lymphovascular invasion may not always be available in a routine frozen section pathology during surgery.

Yu et al. recently reported a nomogram based on the factors which included serum tumor markers such as CA 15–3 and CEA^14^. They demonstrated that the addition of CEA and CA15-3 significantly improved the discrimination ability of the nomogram compared to the nomogram without both serum markers. (AUC: 0.773 (0.732–0.815) vs. 0.727 (0.682–0.771), *p* < 0.001). They also included a number of positive and negative SLNs as two different factors. However, serum CEA levels may be elevated not only in colorectal cancer but other types of cancers. CEA may not be a routinely measured biomarker for breast cancer patients in many institutions. And the nomogram was based on patients’ data who underwent upfront surgery with 1–2 positive SLN macrometastasis so that its use is limited for patients who were treated with preoperative systemic therapy.

Jeruss et al. suggested a model for predicting the likelihood of NSLNM(s) in patients with a positive SLN after NAC^6^. They included five clinicopathologic factors: method of detection of SLN metastasis, multicentricity, initial lymph node status, pathologic tumor size and lymphovascular invasion. The AUC of this model was 0.85, and the bootstrap corrected AUC was 0.76. Their study population was a group of patients with only one metastatic sentinel node who underwent NAC so that application of this nomogram to patients with more than one nodal disease burden may have limited value. The variables of this nomogram also involved pathologic tumor size and lymphovascular invasion which were overlapped with the previous prediction model suggested by Van Zee et al. Both parameters may only be available in a permanent pathology report in many institutions.

Another prediction model of NSLNM for patients who received NAC was suggested by Ryu et al.^12^ They generated a nomogram composed of four variables: pathologic T stage, lymphovascular invasion, SLN metastasis size, and number of positive SLN metastases. Their nomogram exhibited the AUC of 0.791 for internal validation while the AUC for external validation cohort was 0.705. The numbers of included patients were 197 for the developing cohort and 30 for the external validation, respectively. Our study analyzed 384 patients’ data to develop the nomogram and we tested the formula with 174 patients’ data from different time window. Besides the difference of the volume of included patients, their data involved 57 patients who were SLN negative but underwent ALND. Whereas our study included only the patients with 1- 3 positive SLN(s) for analysis.

The forementioned studies and this study share a common purpose of accurate prediction of NSLNM that may provide a guidance for a surgeon to be more selective for finding full-extent ALND candidates. But de-escalation of axillary surgery to the eligible patients might raise a concern of possible residual metastatic nodal disease and its associated risk of tumor recurrence in the future. Nguyen et al. reported a significant shift in axillary surgery trend for clinical N1 patients treated with NAC, with increasing use of SLN surgery while decreasing use of ALND^15^. Although de-escalation of axillary surgery after NAC has been an increasing trend, significant prospective data regarding disease recurrence and related survival are lacking^16^.

A retrospective study compared the survival result between SLNB alone and full extent ALND in patients with 1–3 positive sentinel nodes on intraoperative frozen biopsy after NAC^17^. At a median follow-up of 59.4 months for 483 patients (SLNB alone 188 and ALND 295, respectively), they reported no significant survival difference between the two groups of patients. Their analysis suggested that limited axillary surgery may be a possible surgical option for selected eligible patients.

On the other hand, Almahariq et al. reported that SLNB alone was associated with significantly lower survival than ALND group. (HR 1.7, 95% CI 1.3–2.2, *p* < 0.001), with estimated 5-year overall survival of 71% in SLNB only group compared with 77% of ALND group. (*p* = 0.01) when they compared the survival of a total of 1617 eligible ypN1 patients in the National Cancer Database^18^. However, they found that SLNB may have comparable result with ALND in the selected patients with luminal A or B tumors with a single metastatic lymph node disease. (HR 1.03, 95% CI 0.59–1.8, *p* = 0.91) They had a cautious perspective about reducing the extent of axillary surgery but they also showed limiting the axillary surgery might be feasible to some selected patients with favorable tumor biology. Until further confirmative clinical data is published, our perspective would be that more cautious approach to ypN1 breast cancer patients would still be appropriate but it is worth trying to be more selective in choosing the eligible patients for reduced range of axillary management.

This study has several limitations. Regarding the single institutional, retrospective nature of this study, we would admit that potential selection bias of eligible patients might exist. There were observed heterogeneity among baseline patients’ characteristics between the test group and validation group. The proportion of clinical N0 patients were more included in the baseline group whereas the proportion of clinical N2 patients were higher in the validation cohort. However, throughout the entire patients’ data analysis, we could observe significant differences in the baseline clinicopathologic factors between the cohort with non-sentinel node metastasis and the cohort without residual nodal disease. Also, it is meaningful to show that this is the data that most reflects the real practice that shows the recent trend of changes in surgical methods of operators. The resulting nomogram was validated only in a patient cohort from a single institution despite of its different period of treatment time window. External validation with sufficient number of patients and patients with different background demographic data needs to be done to further verify the correlation of this proposed nomogram.

## Conclusions

The ultimate goal would be to tailor appropriate axillary surgery according to each patients’ disease status such that only patients with expected potential benefit from the ALND would be subjected to the possible morbidity. Also we hope to spare patients who may not gain significant benefit from the extensive procedure. This nomogram could offer clinically useful information to a surgeon whether to proceed further axillary dissection for patients who had 1–3 positive SLN(s) after NAC. As a result, we may have additional guiding tool to decide ALND intraoperatively so that a patient may avoid additional separate surgery session of ALND.

## Supplementary Information

Supplementary Table 1.

## Data Availability

The datasets used and analysed during the current study available from the corresponding author on reasonable request.
