# Supplementary material for: Non-sentinel node metastasis prediction during surgery in breast cancer patients with one to three positive sentinel node(s) following neoadjuvant chemotherapy
Source: Sci Rep. 2023 Mar 18;13:4480. doi: 10.1038/s41598-023-31628-2 (PMC10024769; doi:10.1038/s41598-023-31628-2)
Supplement: Supplementary file 1 — Supplementary Table 1. [file 41598_2023_31628_MOESM1_ESM.docx]

Supplementary table 1. Clinicopathologic characteristics of the training group and validation group.

|  |  |  |  |  |
| --- | --- | --- | --- | --- |
|  | Training group | | Validation group | |
|  | Residual nodal disease | | | |
|  | no | yes | no | yes |
| Age |  |  |  |  |
| <50 years | 142 (66.0) | 109 (64.5) | 69 (70.4) | 35 (46.1) |
| ≥50 years | 73 (34.0) | 60 (35.5) | 29 (29.6) | 41 (53.9) |
| number of positive SLNs |  |  |  |  |
| 1 | 123 (57.2) | 67 ( 39.6) | 62 (63.3) | 32 (42.1) |
| 2 | 72 (33.5) | 72 (42.6) | 34 (34.7) | 25 (32.9) |
| 3 | 20 (9.3) | 30 (17.8) | 2 (2.0) | 19 (25.0) |
| invasion depth of SLNs |  |  |  |  |
| mean(mm) ± SD | 4.14 ± 2.9 | 5.60 ± 3.9 | 5.24 ± 2.8 | 5.68 ± 3.9 |
| number of frozen SLNs |  |  |  |  |
| 1 | 7 (3.3) | 14 (8.3) | 0 (0.0) | 5 (6.6) |
| 2 | 21 (9.8) | 23 (13.6) | 8 (8.2) | 16 (21.1) |
| 3 | 48 (22.3) | 37 (21.9) | 16 (16.3) | 17 (22.4) |
| 4 | 39 (18.1) | 29 (17.2) | 32 (32.7) | 16 (21.1) |
| 5 | 58 (27.0) | 40 (23.7) | 25 (25.5) | 12 (15.8) |
| 6 | 22 (10.2) | 20 (11.8) | 12 (12.2) | 5 (6.6) |
| 7 | 16 (7.4) | 5 (3.0) | 4 (4.1) | 3 (3.9) |
| 8 | 1 (0.5) | 1 (0.6) | 1 (1.0) | 2 (2.6) |
| 9 | 2 (0.9) | 0 (0.0) | ㅡ | ㅡ |
| 11 | 1 (0.5) | 0 (0.0) | ㅡ | ㅡ |
| Tumor grade |  |  |  |  |
| G1/2 | 166 (77.2) | 147 (87.0) | 76 (77.6) | 67 (88.2) |
| G3 | 49 (22.8) | 22 (13.0) | 22 (22.4) | 9 (11.8) |
| Estrogen Receptor |  |  |  |  |
| negative | 54 (25.1) | 22 (13.0) | 19 (19.4) | 13 (17.1) |
| positive | 161 (74.9) | 147 (87.0) | 79 (80.6) | 63 (82.9) |
| Progesterone Receptor |  |  |  |  |
| negative | 83 (38.6) | 42 (24.9) | 36 (36.7) | 26 (34.2) |
| positive | 132 (61.4) | 127 (75.1) | 62 (63.3) | 50 (65.8) |
| HER2 status |  |  |  |  |
| negative | 162 (75.3) | 136 (80.5) | 79 (80.6) | 60 (78.9) |
| positive | 53 (24.7) | 33 (19.5) | 19 (19.4) | 16 (21.1) |
| Biological Subtype |  |  |  |  |
| HR+/ HER2- | 136 (63.3) | 124 (73.4) | 63 (64.3) | 56 (73.7) |
| HR+/ HER2+ | 25 (11.6) | 24 (14.2) | 16 (16.3) | 7 (9.2) |
| HR-/ HER2+ | 27 (12.6) | 8 (4.7) | 3 (3.1) | 6 (7.9) |
| HR-/ HER2- | 27 (12.6) | 13 (7.7) | 16 (16.3) | 7 (9.2) |
| Initial T stage |  |  |  |  |
| 1 | 20 (9.3) | 12 (7.1) | 12 (12.2) | 10 (13.2) |
| 2 | 145 (67.4) | 100 (59.2) | 63 (64.3) | 46 (60.5) |
| 3 | 43 (20.0) | 52 (30.8) | 23 (23.5) | 20 (26.3) |
| 4 | 7 (3.3) | 5 (3.0) | 0 (0.0) | 0 (0.0) |
| Initial N stage |  |  |  |  |
| 0 | 51 (23.7) | 19 (11.2) | 14 (14.3) | 8 (10.5) |
| 1 | 155 (72.1) | 131 (77.5) | 74 (75.5) | 55 (72.4) |
| 2 | 9 (4.2) | 19 (11.2) | 10 (10.2) | 13 (17.1) |
| Surgery type |  |  |  |  |
| Breast Conserving Surgery | 105 (48.8) | 67 (39.6) | 50 (51.0) | 36 (47.4) |
| Mastectomy | 110 (51.2) | 102 (60.4) | 48 (49.0) | 40 (52.6) |
| Response to NAC |  |  |  |  |
| Complete remission | 12 (5.6) | 6 (3.6) | 2 (2.0) | 2 (2.6) |
| Partial remission | 149 (69.3) | 120 (71.0) | 79 (80.6) | 58 (76.3) |
| Stable disease | 50 (23.3) | 40 (23.7) | 15 (15.3) | 14 (18.4) |
| Progressive disease | 4 (1.9) | 3 (1.8) | 2 (2.0) | 2 (2.6) |
| Pathologic T stage |  |  |  |  |
| 0 | 14 (6.5) | 6 (3.6) | 4 (4.1) | 5 (6.6) |
| 1 | 104 (48.4) | 60 (35.5) | 46 (46.9) | 25 (32.9) |
| 2 | 86 (40.0) | 78 (46.2) | 38 (38.8) | 36 (47.4) |
| 3 | 11 (5.1) | 25 (14.8) | 10 (10.2) | 10 (13.2) |
| Pathologic N stage |  |  |  |  |
| 1 | 213 (99.1) | 76 (45.0) | 96 (98.0) | 34 (44.7) |
| 2 | 2 (0.9) | 93 (55.0) | 2 (2.0) | 42 (55.3) |
| SLN: sentinel lymph node |  |  |  |  |
| ER score, PR score: preoperative result. | |  |  |  |
| NAC: Neoadjuvant chemotherapy | |  |  |  |
